# Challenges and Perspectives of Neurological Disorders

**DOI:** 10.3390/brainsci13040676

**Published:** 2023-04-18

**Authors:** Dina Nur Anggraini Ningrum, Woon-Man Kung

**Affiliations:** 1 Public Health Department, Faculty of Sport Sciences, Universitas Negeri Semarang, Semarang 50229, Indonesia; 2 Division of Neurosurgery, Department of Surgery, Taipei Tzu Chi Hospital, Buddhist Tzu Chi Medical Foundation, New Taipei City 23142, Taiwan; 3 Department of Exercise and Health Promotion, College of Kinesiology and Health, Chinese Culture University, Taipei 11114, Taiwan

Neurological disorders pose significant challenges to healthcare systems worldwide. These conditions can severely impact an individual’s quality of life, leading to physical, emotional, and cognitive impairments [1]. Managing neurological disorders often requires specialized care, including access to medical experts, various diagnostic tools, and complicated treatment options. Unfortunately, most real-world scenarios lack sufficient resources to provide adequate care to patients with neurological disorders. Furthermore, the complexity of these conditions makes diagnosis and treatment difficult, leading to misdiagnosis and delayed care, which can exacerbate symptoms and increase the burden on patients and caregivers [2]. Addressing these challenges requires a comprehensive approach that includes improving access to care, investing in research to advance diagnostic and treatment options, and increasing public awareness of neurological disorders [3]. The articles contained in this Special Issue highlight significant advances and encourage further investigational efforts in this exciting field.

The hereditary etiology of neuronal intranuclear inclusion disease (NIID) and its existence in other neurodegenerative disorders is an interesting theme. A study by Wan et al. screened 476 individuals with amyotrophic lateral sclerosis (ALS) and 210 individuals without ALS for the manifestation of a GGC repeat expansion in the Notch Homolog 2 N-terminal-like C gene (NOTCH2NLC). The outcomes indicated that intermediate NOTCH2NLC GGC repeat expansion was connected with Chinese patients with ALS [4].

Zhou et al. reported a rare case report of an individual with NIID who presented with mitochondrial encephalomyopathy, lactic acidosis, and stroke-like (MELAS-like) symptoms, as well as reversible brain magnetic resonance imaging (MRI) diffusion-weighted imaging (DWI) hyperintensities. The diagnosis of this presented case was determined through skin biopsy in addition to genetic testing, in which a steroid treatment resulted in improved symptoms and neuroimaging. This article emphasizes the importance of distinguishing NIID from MELAS and the potential for reversible DWI hyperintensities in NIID [5].

Avramouli’s laboratory analyzed the role of proteolipid protein (PLP) 1 missense point mutations in the pathogenicity of multiple sclerosis (MS). Computational structural biology methods were applied for the evaluation of these mutation effects on the structural stability and flexibility of PLP1. This study demonstrated that the vast majority of variants can change the functionality of protein structures, and in silico genomic methods were likewise carried out to predict the importance of these mutations related to protein functionality. The study suggests that a better description of therapeutic applications and clinical strategies in patients with MS can be achieved by further research into the impact of these mutations [6].

Jiao et al. used radiomics analysis to improve classification accuracy in individuals with Alzheimer’s disease (AD) and mild cognitive impairment (MCI). They aimed to identify high-order features from pathological biomarkers and to improve classification accuracy based on tau positron emission tomography (PET) images. Distinct cohorts were used in the study, and the radiomics features of tau PET imaging of AD-related brain regions were computed for classification using a support vector machine (SVM) model. The model was trained and validated in the first cohort and tested in the second. The results showed that Tau PET radiomics analysis offers a perspective to anticipate clinical diagnosis as well as to figure out risk factors in MCI patients [7].

The study proposed by Jiang et al. features a new machine learning (ML) analytical method that utilizes electroencephalography (EEG), eye tracking (ET), and neuropsychological assessments to screen for MCI in the community. The proposed model achieved high classification accuracy in both training and validation groups and in an independent test group. The proposed model also provided exceptional classification performances, advocating its capacity for subsequent use in predicting cognitive decline [8].

Besides the previously mentioned ML approach, this study proposed another novel model utilizing deep learning radiomics (DLR) by Zhao et al. to differentiate AD, MCI, and normal control (NC) subjects by tau PET scans. The DLR model performed the most outstanding classification performance, when compared to traditional models, thus demonstrating potential clinical value in discriminating AD, MCI, and NC [9].

Notably, the application of a new second-generation tau radiotracer, ^18^F-Florzolotau, was investigated to estimate the association of regional tau accumulation and brain functional connectivity (FC) abnormalities in patients with AD and MCI. Additionally, the proportion loss of functional connectivity strength (PLFCS) was found to be a new indicator of brain FC alteration. In the research performed by Ju et al., the authors found that PLFCS and functional connection strength (FCs) were higher in the AD and MCI groups when compared to the normal control group. The study concludes that brain FC abnormality is correlated with tau pathology in AD and MCI [10].

In a clinical study, Lee et al. implied that natural killer activity (NKA) was significantly impaired in glioblastoma patients, but it recovered and was significantly enhanced on postoperative day (POD) 30, particularly in patients who underwent gross total resection (GTR) when compared to those who underwent subtotal resection (STR). The impaired NKA recovery was also associated with an increase in the CD56^bright^CD16^-^ NK cell subset. Therefore, the study suggests that GTR may improve NKA and increase the CD56^bright^CD16^-^ NK subset, which could be associated with subsequent patient prognosis, and should be performed when possible [11].

In the following research by Yi et al., the association of residual stenosis severity or reperfusion status with artery reocclusion following endovascular treatment for patients with middle cerebral artery (MCA) atherosclerotic ischemic occlusions was inspected. The authors showed that reperfusion status was significantly associated with intraprocedural reocclusion, and individuals experiencing effective thrombectomy reperfusion had a smaller proportion of intraprocedural occlusion regardless of residual stenosis severity. Moreover, once effective reperfusion was attained, the delayed reocclusion rate was relatively decreased and did not significantly differ between individuals with severe residual stenosis and patients with mild to moderate residual stenosis [12].

Riso’s team investigated Erdheim–Chester disease (ECD), an unusual clonal disorder of histiocytic myeloid precursors depicted by multisystem involvement, and its neurological presentations. They retrospectively collected and described a small number of patients with ECD, all revealing cerebellar presentations. The ECD clinical neurological manifestation always includes cerebellar features, demonstrating a subacute or progressive course. The study suggests that recognizing ECD can be extremely challenging with certain unique expressions that are beneficial for addressing it [13].

Finally, it is important to notice that the accessibility of virtual reality (VR) technology for people with neurological disorders has not been explored extensively. An innovative perspective communication by Moon et al. suggests that future research should focus on expanding the use of VR technology for diagnostic purposes and studying its potential benefits in neurological disorders [14].

In summary, these articles reflect recent advances and explore the use of innovative technologies and techniques in the field of neurology. We aim to explore an updated advancement of scientific knowledge to improve patient care. In hope that this collection will not only stimulate further research studies, we expect researchers and clinicians to facilitate the importance of making these tools accessible to individuals with neurological disorders.
